# A Novel and Validated Inflammation-Based Score (IBS) Predicts Survival in Patients With Hepatocellular Carcinoma Following Curative Surgical Resection: A STROBE-Compliant Article

**DOI:** 10.1097/MD.0000000000002784

**Published:** 2016-02-18

**Authors:** Yi-Peng Fu, Xiao-Chun Ni, Yong Yi, Xiao-Yan Cai, Hong-Wei He, Jia-Xing Wang, Zhu-Feng Lu, Xu Han, Ya Cao, Jian Zhou, Jia Fan, Shuang-Jian Qiu

**Affiliations:** From the Liver Cancer Institute, Zhongshan Hospital and Shanghai Medical School (Y-PF, X-CN, YY, X-YC, H-WH, J-XW, Z-FL, JZ, JF, S-JQ) and Biomedical Research Center, Zhongshan Hospital (XH, S-JQ), Fudan University; Key Laboratory for Carcinogenesis and Cancer Invasion, The Chinese Ministry of Education (Y-PF, X-CN, YY, X-YC, H-WH, J-XW, Z-FL, JZ, JF, S-JQ), Shanghai, P.R. China; and Cancer Research Institute, Xiangya School of Medicine, Central South University (YC), Hunan, China.

## Abstract

As chronic inflammation is involved in the pathogenesis and progression of hepatocellular carcinoma (HCC), we investigated the prognostic accuracy of a cluster of inflammatory scores, including the Glasgow Prognostic Score, modified Glasgow Prognostic Score, platelet to lymphocyte ratio, Prognostic Nutritional Index, Prognostic Index, and a novel Inflammation-Based Score (IBS) integrated preoperative and postoperative neutrophil to lymphocyte ratio in 2 independent cohorts. Further, we aimed to formulate an effective prognostic nomogram for HCC after hepatectomy.

Prognostic value of inflammatory scores and Barcelona Clinic Liver Cancer (BCLC) stage were studied in a training cohort of 772 patients with HCC underwent hepatectomy. Independent predictors of survival identified in multivariate analysis were validated in an independent set of 349 patients with an overall similar clinical feature.

In both training and validation cohorts, IBS, microscopic vascular invasion, and BCLC stage emerged as independent factors of overall survival (OS) and recurrence-free survival (RFS). The predictive capacity of the IBS in both OS and RFS appeared superior to that of the other inflammatory scores in terms of C-index. Additionally, the formulated nomogram comprised IBS resulted in more accurate prognostic prediction compared with BCLC stage alone.

IBS is a novel and validated prognostic indicator of HCC after curative resection, and a robust HCC nomogram including IBS was developed to predict survival for patients after hepatectomy.

## Abstract 2

Supplemental Digital Content is available in the text

## INTRODUCTION

Hepatocellular carcinoma (HCC) is the fifth most frequently diagnosed cancer, and the second leading cause of cancer-related deaths worldwide.^1^ Although curative resection is applicable in selected candidates, 60% to 70% patients developed metastasis and recurrence within 5 years following surgery even with radical resection.^2^

Distinct from other solid cancers, the prognosis for patients with HCC rely not solely on the tumor progression but also on the extent of liver dysfunction owing to that approximately 70% to 90% HCCs arise in the context of chronic liver inflammation and cirrhosis.^3,4^ Consequently, staging systems such as tumor node metastasis that depend solely on pathological characteristics retain limited prognostic impact in HCC.^5,6^ A number of alternative systems have been proposed for HCC from independent groups, including the Barcelona Clinic Liver Cancer (BCLC),^7^ Cancer Liver Italian Program,^8^ the Chinese University Prognostic Index,^9^ and Japanese Integrated Score (JIS).^10^ However, there is no worldwide consensus on which is the best system in staging and predicting the outcome of patients with HCC.^11^ Thus, a convenient and reliable Prognostic Index (PI) that can be applied in routine clinical practice is urgently needed.

Inflammation has emerged as the seventh hallmark of cancer,^12^ and accumulating evidence indicated that the presence of systemic inflammation response is associated with poor survival in various malignancies including HCC.^13^ Various markers of systemic inflammation response, including C-reactive protein (CRP),^14^ cytokines,^13^ and absolute count of blood neutrophil or lymphocyte as well as their ratio (NLR) have been explored for their prognostic impact in certain cancer populations.^15–18^

An elevated NLR has been reported to be a predictor of poor survival in patients with HCC underwent hepatic resection,^19,20^ radio-frequency ablation (RFA),^21,22^ transcatheter arterial chemoembolization (TACE),^23–25^ and liver transplantation.^26–28^ NLR may fluctuate in the influence of systemic inflammation response induced by surgical procedure in local. However, these studies only focused on pretreatment NLR change, while the post-treatment NLR change, which may dynamic reflect the change of balance between host inflammatory response and immune response following therapy is largely neglected. In addition, in most of those studies, the cutoff value of NLR has been set empirically. As a consequence, the consensus on cutoff value accurately predicting outcome in patients with HCC is far from achieved.

The integration of serum CRP and albumin has previously been applied to formulate the Glasgow Prognostic Score (GPS)^29^ as an indicator of systemic inflammation. The prognostic impact of this system has been qualified in various solid tumors, including lung,^30^ ovarian,^31^ gastro-esophageal,^32^ colorectal,^33^ and HCC.^34^ Further, accumulating studies have demonstrated that inflammation-based scores (IBSs) such as the modified Glasgow Prognostic Score (mGPS)^35^ and the Prognostic Nutritional Index (PNI),^36^ are associated with prognosis in patients with HCC. Furthermore, the platelet to lymphocyte ratio (PLR)^37^ was as well identified as a significant prognostic indicator in patients with pancreatic cancer. In addition, an integration of serum CRP and white cell count as the PI^38^ was also demonstrated as a significant prognostic predictor in patients with lung cancer. Therefore, which IBS is more accurate for predicting prognosis in patients with HCC remains to be elucidated.

Nomograms are statistical models that specifically developed to optimize predictive accuracy of individuals. While other predictive models assign prognosis based on risk groups, nomograms provide a more individualized prediction of outcome based on a combination of variables. Currently, nomograms have been established in various cancer types.^39,40^ The predictive accuracy of nomograms has compared superior to the traditional staging systems in many cancer populations, and thus, it has been proposed as an alternative or even as a new standard.^41^ Given the individualized predictive capacity of this statistical tool, further establishing a nomogram comprised IBS is significant.

The goals of the present research were to evaluate the prognostic capacity of a novel defined IBS that combine the preoperative and postoperative NLR in 2 independent cohorts. Further, we aimed to compare its accuracy with routinely clinical used prognostic model BCLC stage together with other systemic inflammation scores such as GPS, mGPS, PNI, PLR, and PI to ascertain whether IBS is a feasible prognostic indicator. In addition, we wished to develop a prognostic nomogram including IBS in patients with HCC after curative resection.

## MATERIALS AND METHODS

### Patients

Two independent cohorts of patients with HCC after curative resection were enrolled in this study. In brief, after obtaining Ethics Committee approval, we used computer-generated random numbers via SPSS software (SPSS Inc., Chicago, IL) and randomly selected 772 patients from HCC patients underwent surgical resection from December 2010 to June 2012 in Zhongshan Hospital as training cohort and 349 patients from HCC patients underwent surgical resection in 2007 as validation cohort. All the patients had survived for at least 30 days postoperatively. The eligibility criteria for the patients analyzed in both training and validation cohorts are as follows: all patients with an exact pathological diagnosis of HCC; all patients underwent resection defined as a complete resection of all tumor lesions and the cut surface being free of cancer by histological examination; all patients’ records included complete clinicopathologic and follow-up data; all the blood samples were obtained within 3 days before operations and on the 5th day following resection; cases with a history of inflammation disease or active concomitant infection were excluded; and patients received preoperative anticancer treatments were all excluded in case of any bias. The study protocol was approved by the Ethics Committee of Zhongshan Hospital affiliated to Fudan University, and each patient provided informed consent to participate in the study.

### Follow-Up

The follow-up procedure was described in our previous study^42^; in brief, all patients were followed up every 3 months during the first postoperative year and at least 3 to 6 months thereafter. α-fetoprotein (AFP), liver function test, chest X-ray, and liver ultrasonography were performed during each follow up. Once recurrence was suspected, whether intrahepatic recurrence or distal metastasis had occurred was further validated via computed tomography and/or magnetic resonance imaging. The period from the time of resection to the time of either death or last follow-up was defined as overall survival (OS). Recurrence-free survival (RFS) was calculated as the period between operation and time of recurrence. If recurrence was not found, the RFS was calculated from the time of surgery to the time of death or last follow-up. The median follow-up time was 39 months (range, 2–60 months) in training cohort and 43 months (range, 1.5–72 months) in validation cohort.

### Inflammation-Based Prognostic Scores and Other Variables

Blood samples were collected within 3 days before surgery as well as on the fifth postoperative day. CRP, bilirubin, albumin, alanine aminotransferase (ALT), aspartate aminotransferase (AST), complete blood count, and BCLC scores were obtained. The inflammation-based prognostic score were formulated as detailed in Table 1.

**TABLE 1:**
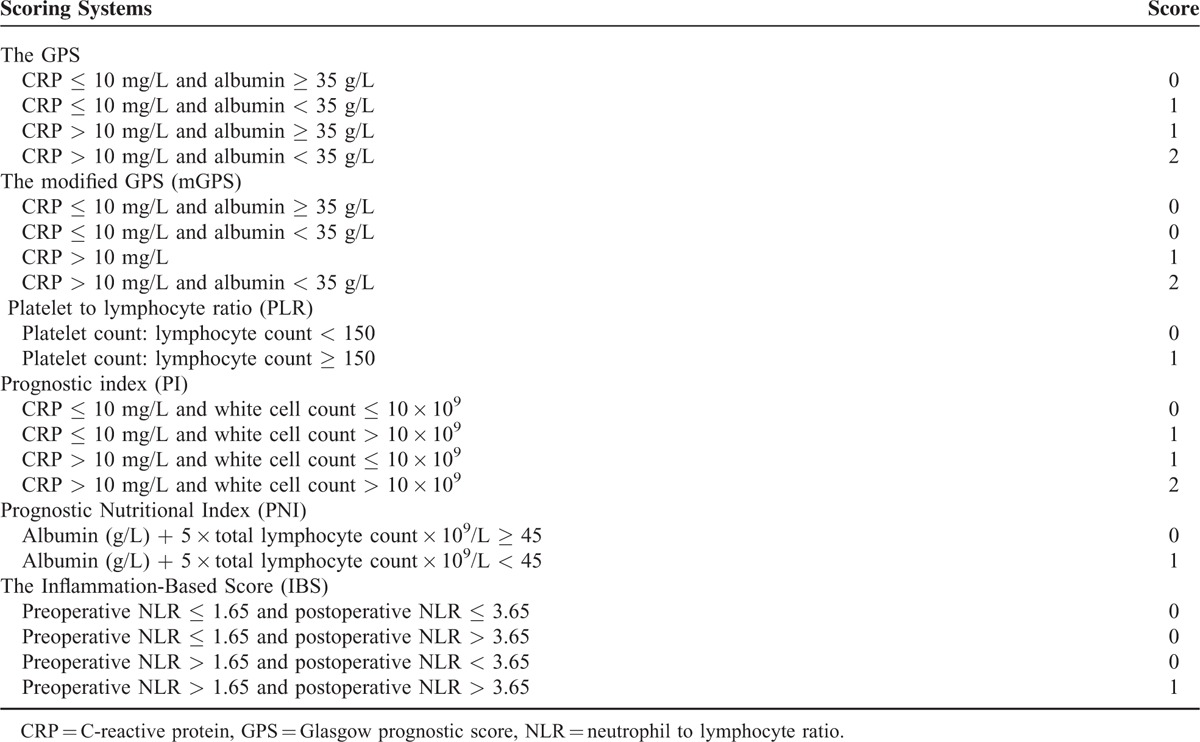
Inflammation-Based Prognostic Scores

### Statistical Analysis

Statistical analysis was performed via SPSS version 16 (SPSS Inc). The Mann–Whitney *U* test was used for comparison between 2 independent groups. Any associations between variables were analyzed using Pearson Chi-squared test. Survival curves were depicted via the Kaplan–Meier method and compared using the log-rank test. Univariate and multivariate analyses for the prognostic factors were based on the Cox proportional hazard model. A nomogram was formulated based on the results of multivariate analysis, and C-index was generated to evaluate the discriminatory ability of each scoring systems.

## RESULTS

### Optimal Cut-Off Value for Elevated NLR

The optimal cut-off value of preoperative and postoperative NLR for recurrence and death prediction was determined via receiver operating characteristic (ROC) curve analysis. Preoperative and postoperative NLR were associated with the strongest Youden index for both cancer-related death and recurrence prediction with a cutoff value of 1.65 and 3.65, respectively. For cancer-related death prediction, the area under the ROC curve was 0.631 (95% confidence interval [CI], 0.583–0.679) for preoperative NLR and 0.568 (95% CI, 0.517–0.628) for postoperative NLR (Supplementary Figure S1A). For recurrence prediction in the training cohort, the area under the ROC curve was 0.576 (95% CI, 0.531–0.616) and 0.562 (95% CI, 0.522–0.617) for preoperative and postoperative NLR, respectively (Supplementary Figure S1B).

### Relationship Between Inflammatory Scores and Patient Characteristics

The relationship between inflammatory scores and clinicopathological features of the training cohort is summarized in Supplementary Table S1. A raised IBS was associated with larger tumor size, presence of microscopic vascular invasion, and advanced BCLC stage. GPS, mGPS, and PI were all linked with raised gamma-glutamyl transpeptidase (GGT), larger tumor diameter, presence of microscopic vascular invasion, and advanced BCLC stage. PNI was associated with poor cancer cell differentiation and elevated PLR was a significant indicator of multiple tumor number.

### Inflammatory Scores and Survival in the Training Cohort

**FIGURE 1:**
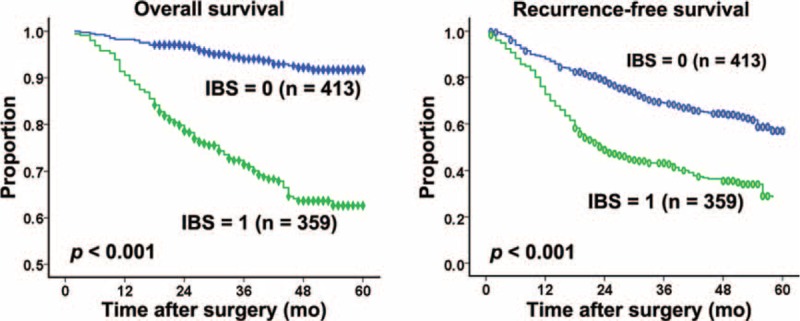
Kaplan–Meier survival curves for patients in training cohort stratified by IBS. (A) Overall survival and (B) recurrence-free survival. IBS = Inflammation Based Score.

The follow-up was completed on November 10, 2015, with median follow-up time of 39 months (range, 2–60), the 1-, 3-, 4-year OS and RFS rates were 88.2%, 59.9%, 51.2% and 79.9%, 59.4%, 45.4%, respectively. In univariate analysis, elevated IBS (*P* < 0.001) (Figure 1A), PLR (*P* = 0.001), PI (*P* < 0.001), GPS (*P* < 0.001), mGPS (*P* < 0.001), raised AFP (*P* < 0.001), GGT (*P* < 0.001), lager tumor size (*P* < 0.001), multiple tumor number (*P* = 0.001), presence of microscopic vascular invasion (*P* < 0.001), poor cancer cell differentiation (*P* < 0.001), and advanced BCLC stage (*P* < 0.001) were identified as significant predictors of OS. The survival rate at the end of the follow-up period were 93.1% and 68.5%; 83% and 68.2%; 84% and 62%; 84.1%, 68.1%, and 44.4%; 84.5%, 63.2%, and 37.5% for patients with IBS of 0 and 1; with PLR of 0 and 1; with PI of 0 and 1; with GPS of 0, 1, and 2; with mGPS of 0, 1, and 2, respectively. In multivariate analysis, IBS (hazard ratio [HR], 4.247, 95% CI, 2.786–6.473; *P* < 0.001), mGPS (HR, 3.508, 95% CI, 1.384–8.890; *P* = 0.008), GGT (HR, 1.699, 95% CI, 1.192–2.422; *P* = 0.003), tumor numbers (HR, 2.152, 95% CI, 1.444–3.205; *P* < 0.001), presence of microscopic vascular invasion (HR, 2.363, 95% CI, 1.637–3.413; *P* < 0.001), and BCLC stage (HR, 1.634, 95% CI, 1.225–2.197, *P* = 0.001) remained as significant independent predictors of OS (Table 2).

**TABLE 2:**
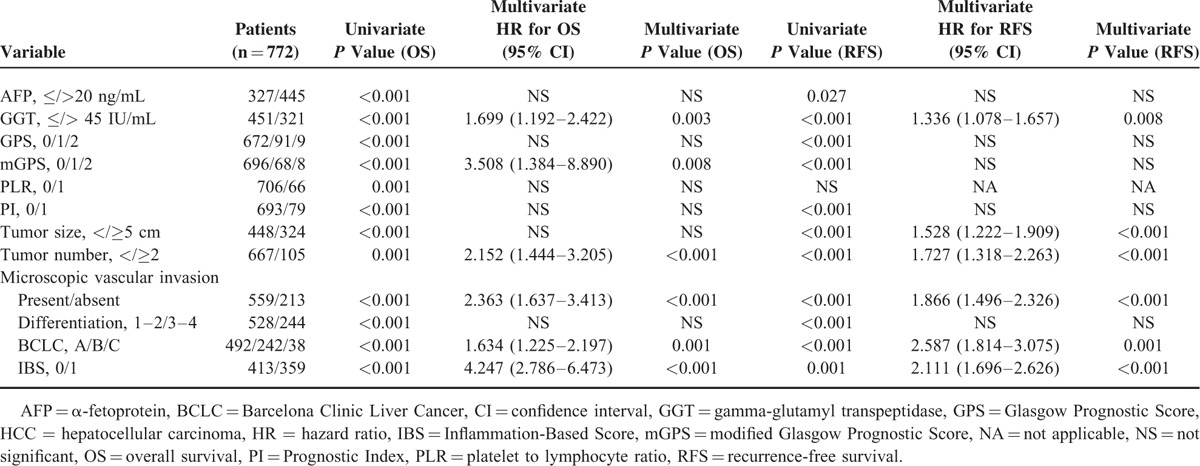
Clinicopathological Characteristics in Patients With HCC: Univariate and Multivariate Survival Analyses (Training Cohort)

In univariate analysis, elevated IBS (*P* = 0.001) (Figure 1B), PI (*P* < 0.001), GPS (*P* < 0.001), mGPS (*P* < 0.001), raised AFP (*P* = 0.027), GGT (*P* < 0.001), lager tumor size (*P* < 0.001), multiple tumor number (*P* < 0.001), presence of microscopic vascular invasion (*P* < 0.001), poor cancer cell differentiation (*P* < 0.001), and advanced BCLC stage (*P* < 0.001) were identified as significant predictors of RFS. The RFS rate at the end of the follow-up period were 65.9% and 38.78%; 55.3% and 35.4%; 55.8%, 44.4%, and 35.2%; 55.2, 37.5%, and 35.3% for patients with IBS of 0 and 1; with PI of 0 and 1; with GPS of 0, 1, and 2; with mGPS of 0, 1, and 2, respectively. In multivariate analysis, IBS (HR, 2.111, 95% CI, 1.969–2.626; *P* < 0.001), GGT (HR, 1.336, 95% CI, 1.078–1.657; *P* = 0.008), presence of microscopic vascular invasion (HR, 1.866, 95% CI, 1.496–2.326; *P* < 0.001), and BCLC stage (HR, 2.587, 95% CI, 1.814–3.075; *P* = 0.001) remained as significant independent predictors of RFS (Table 2).

### Comparative Performance of Inflammation-Based Prognostic Scores and BCLC Stage

The discriminatory capacity of each inflammatory scoring system as well as BCLC stage was compared by means of Harrell concordance index. The C-index was calculated for each prognostic system, as shown in Supplementary Tables S2 and S3. For OS prediction, improvement of the discriminatory ability was observed in the prognostic model integrated IBS and BCLC stage, giving rise to a new C-index of 0.777 (95% CI, 0.775–0.779) compared with the previous value of 0.693 (95% CI, 0.691–0.695) for the BCLC stage alone. Similarly, for RFS prediction, improvement of the discriminatory capacity of prognostic model was obtained by integrated IBS into BCLC stage, giving rise to a new C-index of 0.684 (95% CI, 0.682–0.686) compared with the previous value of 0.615 (95% CI, 0.613–0.617) for the BCLC stage alone. The BCLC stage is superior to the inflammatory scoring systems in terms of discriminatory capacity for OS and RFS prediction. The IBS is consistently with a higher C-index value in both OS and RFS prediction in comparison with other inflammation-based prognostic scores (Supplementary Tables S2 and S3).

### Validation of Prognostic Models

The IBS and BCLC stage were further assessed for their prognostic power and discriminative capacity in an independent validation cohort with median follow-up time of 43 months (range, 1.5–72). Comparison of patient clincopathological features across the 2 studies revealed the validation set was composed of smaller proportion of patients with age >55 years old, with ALT <40U/L, with albumin <40 g/L, with poor tumor differentiation, with complete tumor capsule, with tumor larger than 5 cm in size, compared to the training set, as reported in Supplementary Table S4. Of note, in univariate analysis IBS (Supplementary Figure S2) and BCLC stage also showed the ability to stratify patients’ survival. In multivariate analysis, IBS and BCLC stage remained as independent predictors of survival with HR values of 2.547 (95% CI, 1.610–4.029; *P* < 0.001) and 1.802 (95% CI, 1.292–2.512; *P* = 0.001) for OS and 2.732 (95% CI, 1.860–4.011; *P* < 0.001) and 1.594 (95% CI, 1.167–2.177; *P* = 0.003) for RFS, respectively (Supplementary Table S5).

### Prognostic Nomogram for Survival

**FIGURE 2:**
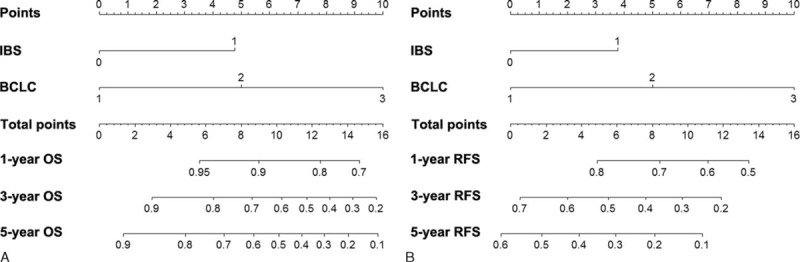
Hepatocellular carcinoma survival nomogram. To use the nomogram, the value of an individual patient is located on each variable axis, and a line is drawn upward to determine the number of points received for each variable value. The sum of these numbers is located on the total point axis, and a line is drawn downward to the survival axes to determine the likelihood of 1-, 3-, and 5-year survival).

**FIGURE 3:**
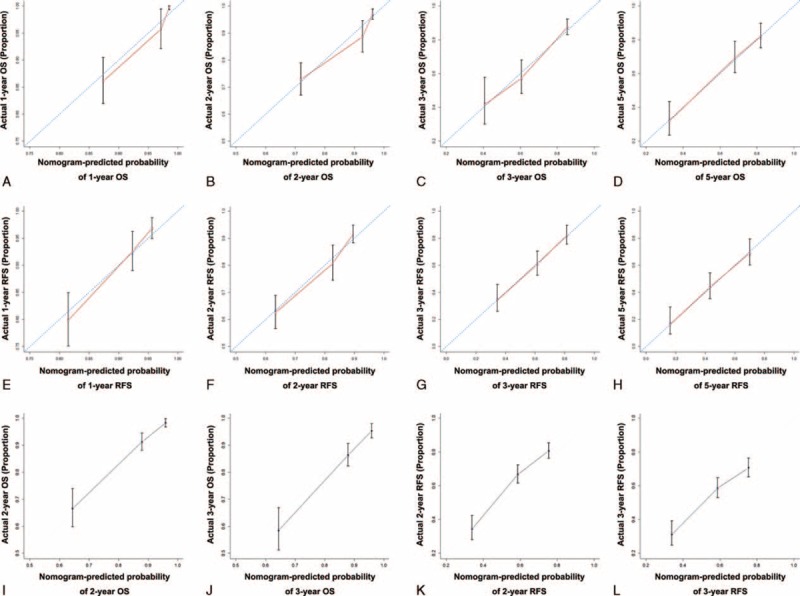
The calibration curve for predicting overall survival of patients at (A) 1 year, (B) 2 year, (C) 3 year, and (D) 5 year; predicting recurrence-free survival at (E) 1 year, (F) 2 year, (G) 3 year, and (H) 5 year in the training cohort; predicting patient overall survival at (I) 2 year and (K) 3 year and predicting patient recurrence-free survival at (J) 2 year and (L) 3 year in the validation cohort. Nomogram-predicted probability of survival is plotted on the x-axis; actual survival is plotted on the y-axis.

The prognostic nomogram that integrated IBS and BCLC stage for OS derived from training cohort is shown in Figure 2A. The C-index for OS prediction in training cohort was 0.777 (95% CI, 0.775–0.779). The calibration plot for the probability of survival at 1, 2, 3, and 5 years after surgery showed optimal consistency between the prediction by nomogram and actual observation (Figure 3A–D).

The prognostic nomogram that combined IBS and BCLC stage for RFS derived from training cohort is shown in Figure 2B. The C-index for RFS prediction in training cohort was 0.684 (95% CI, 0.682–0.686). The calibration plot for the probability of RFS at 1, 2, 3, and 5 years after surgery showed optimal consistency between the prediction by nomogram and actual observation (Figure 3E–H).

### Validation of Prediction Accuracy of the Nomogram for Survival

In the validation set, the median follow-up time was 43 months (range, 1.5–72 months), the median time to recurrence was 35.6 months (range, 1–63 months).

The C-index of the constructed nomogram for predicting OS was 0.699 (95% CI, 0.696–0.702) and a calibration curve fit well between prediction and observation in the probability of both 2-year and 3-year OS (Figure 3I and K). The C-index of the proposed nomogram for predicting RFS was 0.621 (95% CI, 0.619–0.623) and a calibration curve showed good agreement between prediction and observation in the probability of both 2-year and 3-year RFS (Figure 3J and L).

### Comparison of Constructed Nomogram to BCLC Stage as Predictor of OS and RFS on Decision Curve Analysis

**FIGURE 4:**
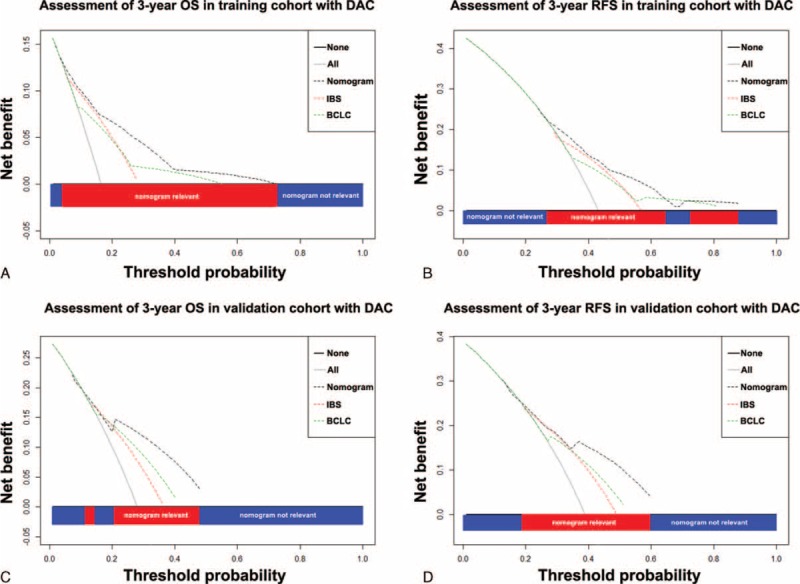
Decision curve analysis. Decision curve analyses depict the clinical net benefit in pairwise comparisons between integrated nomogram and BCLC stage. Nomogram is compared against the BCLC stage in terms of 3-year OS (A and C) and 3-year RFS (B and D) in training and validation cohort, respectively. Dashed lines indicate the net benefit of nomogram in each of the curves across a range of threshold probabilities. The horizontal solid black line represents the assumptions that no patients will experience the event, and the solid gray line represents the assumption that all patients will relapse. On decision curve analysis, nomogram showed superior net benefit compared with BCLC stage across a range of threshold probabilities. BCLC = Barcelona Clinic Liver Cancer, OS = overall survival, RFS = recurrence-free survival.

The nomogram integrated BCLC and IBS demonstrated superior predictive capabilities relative to the BCLC stage alone, with a concordance index of 0.777 (95% CI, 0.775–0.779) for prediction of OS and 0.684 (95% CI, 0.682–0.686) for prediction of RFS. On decision curve analysis,^43^ compared to BCLC stage, our nomogram showed better net benefit with wider range of threshold probability and improved performance for predicting 3-year OS and RFS in training cohort (Figure 4A and B) and 3-year OS and RFS in validation cohort (Figure 4C and D). This represents superior estimation of decision outcomes at higher threshold probability levels.

## DISCUSSION

In this study, we established a novel, easy-to-use, and effective NLR-derived inflammatory score named IBS for predicting outcome in HCC after curative resection. Moreover, a robust HCC nomogram including IBS was developed to improve the predictive power of the current prognostic score.

NLR, a simple and effective marker of inflammation, has shown a significant prognostic value in patients with HCC underwent hepatic resection.^19,20^ However, in most of the studies, the cutoff value of NLR has been set empirically. In addition, these studies only focused on preoperative NLR change, while the postoperative NLR change, which may dynamic reflect the change of balance between host inflammatory and immune response following treatment was largely neglected. In the present study, by using ROC curve analysis, we determined the optimal cutoff value of preoperative NLR was 1.65 and postoperative NLR was 3.65 for prediction of prognosis of patients with HCC after resection.

IBS was constructed via combination of preoperative and postoperative NLR; our study confirmed the findings that an elevated NLR is predictive of poor outcome in HCC patients. Until now, the exact explanation for the observation that elevated NLR in patients with malignancy have worse survival is not clearly defined, but several hypotheses may be accounted for. The neutrophils form important compartment for tumor-related angiogenesis including the level of circulating grow factors, protease, and angiogenesis-regulating chemokines.^44–46^ High infiltration of peritumoral neutrophils were reported to stimulate angiogenesis by releasing matrix metallopeptidase 9 and enhance invasion of tumor cells by production of hepatocyte growth factor, conceivably, their levels could serve as predictor for poor survival in HCC patients.^47^ Conversely, the number of circulating lymphocyte plays critical role in cytotoxic cell death and cytokine production, thus exert inhibition effects on proliferation and metastatic capacity of cancer cells.^48^ The presence of high intratumoral activated CD8 cytotoxic cells was associated with improved survival in HCC.^39^ The impairment of HCC-infiltrating γδ T cells compared with paired peritumoral tissue also indicated poor survival in HCC.^49^ Consequently, when taken together, NLR could reflect the balance between host inflammation and immunity. Raised NLR indicates that the balance is toward the pro-tumor inflammatory response, and is associated with poor prognosis as evidenced by increased intratumoral neutrophils were reported significantly associated with poor survival and intratumoral neutrophil-to-CD8^+^ T cell ratio better predicted the outcome in HCC.^50^

Postoperative NLR change was identified as a significant prognostic factor for tumor recurrence for patients with clear renal cell carcinoma after surgery.^51^ More recently, 2 studies suggested that post-RFA change was also associated with poor survival in HCC.^21,22^ The conveniently measurable and accessible baseline NLR is an inflammatory score with advantage of wide-range continuous data and minimum variation.^52^ Further, the dynamic fluctuation of residual host inflammation activity after tumor resection can be reflected by postoperative NLR. The present study showed that patients whose postoperative NLR was still high predispose worse survival. This suggested that failure of NLR to decrease after tumor resection may serve as surrogate indicator of pro-tumor inflammation after surgery.^53,54^ Otherwise, if NLR remains in a low level after surgery, it indicated that the balance was tipped in conducive to antitumor immune response. More importantly, the strength of prediction seems greater in IBS than that of preoperative NLR alone. In the present study, the preoperative NLR was neither a predictor of OS nor a predictor of RFS, whereas the IBS was a predictor of OS and RFS in both training and validation cohorts.

External validation is preferable in the assessment of novel prognostic indicators in HCC. Importantly, BCLC and IBS stage remain to be identified as predictors of OS and RFS in an independent set of patients with similar tumor burden, indicating the feasibility of these prognostic models. As reported in Supplementary Tables S2 and S3, BCLC stage was found superior to the inflammation-based prognostic scores as well as IBS was superior to the other IBSs for the prediction of prognosis in patients with HCC. The elevated preoperative and postoperative NLRs were included in the IBS as reflection of subclinical inflammation response in favor of tumor progression and decreased antitumor activity, 2 overlapping conditions concerning both preoperative and postoperative status affecting the prognosis of cancer patients.

Some limitations in the present study need to be considered. The first consideration is the different tumor size in the 2 independent cohorts, with a relatively higher rate of tumor size <5 cm in validation cohort compared with that of training cohort; however, the tumor burden in term of BCLC stage is even. Secondly, we did not measure postoperative CRP change together with NLR, because it is not routinely tested in our daily practice. In addition, due to the limited racial and the amount of the patients selected, cross validation and further investigations of IBS in multicenter and prospective setting with more inflammation factors should be conducted in our future researches.

In conclusion, these data indicate that the IBS provides additional prognostic information for the clinical management of HCC patients underwent hepatectomy and the proposed nomogram in this study comprised of IBS objectively and accurately predict the outcome of HCC patients despite the acknowledged limitations. Furthermore, we confirmed that the IBS qualifies as an independent, convenient, and universally available inflammatory score to predict outcome of HCC following surgery.

## Supplementary Material

Supplemental Digital Content

## References

[1] Jemal A Bray F Center MM Global cancer statistics. *CA Cancer J Clin* 2011; 61:69–90. 21296855 10.3322/caac.20107

[2] Llovet JM Burroughs A Bruix J Hepatocellular carcinoma. *Lancet* 2003; 362:1907–1917. 14667750 10.1016/S0140-6736(03)14964-1

[3] Tandon P Garcia-Tsao G Prognostic indicators in hepatocellular carcinoma: a systematic review of 72 studies. *Liver Int* 2009; 29:502–510. 19141028 10.1111/j.1478-3231.2008.01957.x PMC2711257

[4] Schutte K Bornschein J Malfertheiner P Hepatocellular carcinoma—epidemiological trends and risk factors. *Dig Dis* 2009; 27:80–92. 19546545 10.1159/000218339

[5] Zhou L Rui JA Wang SB LCSGJ-T classification, 6th or 5th edition TNM staging did not independently predict the long-term prognosis of HBV-related hepatocellular carcinoma after radical hepatectomy. *J Surg Res* 2010; 159:538–544. 19111323 10.1016/j.jss.2008.09.004

[6] Zhou L Rui JA Ye DX Both the 5th and 6th editions of TNM staging system fail to independently predict long-term prognosis after radical hepatectomy in hepatocellular carcinoma sized > or = 5 cm. *Chin Med Sci J* 2009; 24:220–226. 20120768 10.1016/s1001-9294(10)60005-3

[7] Llovet JM Bru C Bruix J Prognosis of hepatocellular carcinoma: the BCLC staging classification. *Semin Liver Dis* 1999; 19:329–338. 10518312 10.1055/s-2007-1007122

[8] Capuano G Daniele B Gaeta GB A new prognostic system for hepatocellular carcinoma: a retrospective study of 435 patients: the Cancer of the Liver Italian Program (CLIP) investigators. *Hepatology* 1998; 28:751–755. 9731568 10.1002/hep.510280322

[9] Leung TW Tang AM Zee B Construction of the Chinese University Prognostic Index for hepatocellular carcinoma and comparison with the TNM staging system, the Okuda staging system, and the Cancer of the Liver Italian Program staging system: a study based on 926 patients. *Cancer* 2002; 94:1760–1769. 11920539 10.1002/cncr.10384

[10] Kudo M Chung H Haji S Validation of a new prognostic staging system for hepatocellular carcinoma: the JIS score compared with the CLIP score. *Hepatology* 2004; 40:1396–1405. 15565571 10.1002/hep.20486

[11] Camma C Cabibbo G Prognostic scores for hepatocellular carcinoma: none is the winner. *Liver Int* 2009; 29:478–480. 19323777 10.1111/j.1478-3231.2009.01994.x PMC2711259

[12] Hanahan D Weinberg RA Hallmarks of cancer: the next generation. *Cell* 2011; 144:646–674. 21376230 10.1016/j.cell.2011.02.013

[13] Liao R Sun J Wu H High expression of IL-17 and IL-17RE associate with poor prognosis of hepatocellular carcinoma. *J Exp Clin Cancer Res* 2013; 32:3. 23305119 10.1186/1756-9966-32-3 PMC3621615

[14] Polterauer S Grimm C Zeillinger R Association of C-reactive protein (CRP) gene polymorphisms, serum CRP levels and cervical cancer prognosis. *Anticancer Res* 2011; 31:2259–2264. 21737650

[15] Luo G Guo M Liu Z Blood neutrophil-lymphocyte ratio predicts survival in patients with advanced pancreatic cancer treated with chemotherapy. *Ann Surg Oncol* 2014; 22:670–676. 25155401 10.1245/s10434-014-4021-y

[16] Templeton AJ Pezaro C Omlin A Simple prognostic score for metastatic castration-resistant prostate cancer with incorporation of neutrophil-to-lymphocyte ratio. *Cancer* 2014; 120:3346–3352. 24995769 10.1002/cncr.28890

[17] Kang MH Go SI Song HN The prognostic impact of the neutrophil-to-lymphocyte ratio in patients with small-cell lung cancer. *Br J Cancer* 2014; 111:452–460. 24921916 10.1038/bjc.2014.317 PMC4119986

[18] He W Yin C Guo G Initial neutrophil lymphocyte ratio is superior to platelet lymphocyte ratio as an adverse prognostic and predictive factor in metastatic colorectal cancer. *Med Oncol* 2013; 30:439. 23307251 10.1007/s12032-012-0439-x

[19] Gomez D Farid S Malik HZ Preoperative neutrophil-to-lymphocyte ratio as a prognostic predictor after curative resection for hepatocellular carcinoma. *World J Surg* 2008; 32:1757–1762. 18340479 10.1007/s00268-008-9552-6

[20] Fu SJ Shen SL Li SQ Prognostic value of preoperative peripheral neutrophil-to-lymphocyte ratio in patients with HBV-associated hepatocellular carcinoma after radical hepatectomy. *Med Oncol* 2013; 30:721. 24026659 10.1007/s12032-013-0721-6

[21] Dan J Zhang Y Peng Z Postoperative neutrophil-to-lymphocyte ratio change predicts survival of patients with small hepatocellular carcinoma undergoing radiofrequency ablation. *PLoS ONE* 2013; 8:e58184. 23516447 10.1371/journal.pone.0058184 PMC3597630

[22] Chen TM Lin CC Huang PT Neutrophil-to-lymphocyte ratio associated with mortality in early hepatocellular carcinoma patients after radiofrequency ablation. *J Gastroenterol Hepatol* 2012; 27:553–561. 21913982 10.1111/j.1440-1746.2011.06910.x

[23] McNally ME Martinez A Khabiri H Inflammatory markers are associated with outcome in patients with unresectable hepatocellular carcinoma undergoing transarterial chemoembolization. *Ann Surg Oncol* 2013; 20:923–928. 22965570 10.1245/s10434-012-2639-1

[24] Pinato DJ Sharma R An inflammation-based prognostic index predicts survival advantage after transarterial chemoembolization in hepatocellular carcinoma. *Transl Res* 2012; 160:146–152. 22677364 10.1016/j.trsl.2012.01.011

[25] Huang ZL Luo J Chen MS Blood neutrophil-to-lymphocyte ratio predicts survival in patients with unresectable hepatocellular carcinoma undergoing transarterial chemoembolization. *J Vasc Interv Radiol* 2011; 22:702–709. 21514523 10.1016/j.jvir.2010.12.041

[26] Yoshizumi T Ikegami T Toshima T Two-step selection criteria for living donor liver transplantation in patients with hepatocellular carcinoma. *Transplant Proc* 2013; 45:3310–3313. 24182807 10.1016/j.transproceed.2013.05.001

[27] Xiao GQ Liu C Liu DL Neutrophil-lymphocyte ratio predicts the prognosis of patients with hepatocellular carcinoma after liver transplantation. *World J Gastroenterol* 2013; 19:8398–8407. 24363533 10.3748/wjg.v19.i45.8398 PMC3857465

[28] Motomura T Shirabe K Mano Y Neutrophil-lymphocyte ratio reflects hepatocellular carcinoma recurrence after liver transplantation via inflammatory microenvironment. *J Hepatol* 2013; 58:58–64. 22925812 10.1016/j.jhep.2012.08.017

[29] Forrest LM McMillan DC McArdle CS Evaluation of cumulative prognostic scores based on the systemic inflammatory response in patients with inoperable non-small-cell lung cancer. *Br J Cancer* 2003; 89:1028–1030. 12966420 10.1038/sj.bjc.6601242 PMC2376960

[30] Tomita M Ayabe T Chosa E Prognostic significance of pre- and postoperative Glasgow prognostic score for patients with non-small cell lung cancer. *Anticancer Res* 2014; 34:3137–3140. 24922684

[31] Sharma R Hook J Kumar M Evaluation of an inflammation-based prognostic score in patients with advanced ovarian cancer. *Eur J Cancer* 2008; 44:251–256. 18155897 10.1016/j.ejca.2007.11.011

[32] Crumley AB McMillan DC McKernan M Evaluation of an inflammation-based prognostic score in patients with inoperable gastro-oesophageal cancer. *Br J Cancer* 2006; 94:637–641. 16479253 10.1038/sj.bjc.6602998 PMC2361199

[33] Sharma R Zucknick M London R Systemic inflammatory response predicts prognosis in patients with advanced-stage colorectal cancer. *Clin Colorectal Cancer* 2008; 7:331–337. 18794066 10.3816/CCC.2008.n.044

[34] Huang J Xu L Luo Y The inflammation-based scores to predict prognosis of patients with hepatocellular carcinoma after hepatectomy. *Med Oncol* 2014; 31:883. 24535607 10.1007/s12032-014-0883-x

[35] Pinato DJ Stebbing J Ishizuka M A novel and validated prognostic index in hepatocellular carcinoma: the Inflammation Based Index (IBI). *J Hepatol* 2012; 57:1013–1020. 22732513 10.1016/j.jhep.2012.06.022

[36] Pinato DJ North BV Sharma R A novel, externally validated inflammation-based prognostic algorithm in hepatocellular carcinoma: the Prognostic Nutritional Index (PNI). *Br J Cancer* 2012; 106:1439–1445. 22433965 10.1038/bjc.2012.92 PMC3326674

[37] Smith RA Bosonnet L Raraty M Preoperative platelet-lymphocyte ratio is an independent significant prognostic marker in resected pancreatic ductal adenocarcinoma. *Am J Surg* 2009; 197:466–472. 18639229 10.1016/j.amjsurg.2007.12.057

[38] Kasymjanova G MacDonald N Agulnik JS The predictive value of pre-treatment inflammatory markers in advanced non-small-cell lung cancer. *Curr Oncol* 2010; 17:52–58. 20697515 10.3747/co.v17i4.567 PMC2913830

[39] Gao Q Qiu SJ Fan J Intratumoral balance of regulatory and cytotoxic T cells is associated with prognosis of hepatocellular carcinoma after resection. *J Clin Oncol* 2007; 25:2586–2593. 17577038 10.1200/JCO.2006.09.4565

[40] Bochner BH Kattan MW Vora KC Postoperative nomogram predicting risk of recurrence after radical cystectomy for bladder cancer. *J Clin Oncol* 2006; 24:3967–3972. 16864855 10.1200/JCO.2005.05.3884

[41] Sternberg CN Are nomograms better than currently available stage groupings for bladder cancer? *J Clin Oncol* 2006; 24:3819–3820. 16864852 10.1200/JCO.2006.07.1290

[42] Sun HC Zhang W Qin LX Positive serum hepatitis B e antigen is associated with higher risk of early recurrence and poorer survival in patients after curative resection of hepatitis B-related hepatocellular carcinoma. *J Hepatol* 2007; 47:684–690. 17854945 10.1016/j.jhep.2007.06.019

[43] Vickers AJ Elkin EB Decision curve analysis: a novel method for evaluating prediction models. *Med Decis Making* 2006; 26:565–574. 17099194 10.1177/0272989X06295361 PMC2577036

[44] Hung HY Chen JS Yeh CY Effect of preoperative neutrophil-lymphocyte ratio on the surgical outcomes of stage II colon cancer patients who do not receive adjuvant chemotherapy. *Int J Colorectal Dis* 2011; 26:1059–1065. 21479566 10.1007/s00384-011-1192-x

[45] Kusumanto YH Dam WA Hospers GA Platelets and granulocytes, in particular the neutrophils, form important compartments for circulating vascular endothelial growth factor. *Angiogenesis* 2003; 6:283–287. 15166496 10.1023/B:AGEN.0000029415.62384.ba

[46] Fondevila C Metges JP Fuster J p53 and VEGF expression are independent predictors of tumour recurrence and survival following curative resection of gastric cancer. *Br J Cancer* 2004; 90:206–215. 14710231 10.1038/sj.bjc.6601455 PMC2395306

[47] Kuang DM Zhao Q Wu Y Peritumoral neutrophils link inflammatory response to disease progression by fostering angiogenesis in hepatocellular carcinoma. *J Hepatol* 2011; 54:948–955. 21145847 10.1016/j.jhep.2010.08.041

[48] Ding PR An X Zhang RX Elevated preoperative neutrophil to lymphocyte ratio predicts risk of recurrence following curative resection for stage IIA colon cancer. *Int J Colorectal Dis* 2010; 25:1427–1433. 20821217 10.1007/s00384-010-1052-0

[49] Yi Y He HW Wang JX The functional impairment of HCC-infiltrating gammadelta T cells, partially mediated by regulatory T cells in a TGFbeta- and IL-10-dependent manner. *J Hepatol* 2013; 58:977–983. 23262246 10.1016/j.jhep.2012.12.015

[50] Li YW Qiu SJ Fan J Intratumoral neutrophils: a poor prognostic factor for hepatocellular carcinoma following resection. *J Hepatol* 2011; 54:497–505. 21112656 10.1016/j.jhep.2010.07.044

[51] Ohno Y Nakashima J Ohori M Followup of neutrophil-to-lymphocyte ratio and recurrence of clear cell renal cell carcinoma. *J Urol* 2012; 187:411–417. 22177153 10.1016/j.juro.2011.10.026

[52] Azab B Zaher M Weiserbs KF Usefulness of neutrophil to lymphocyte ratio in predicting short- and long-term mortality after non-ST-elevation myocardial infarction. *Am J Cardiol* 2010; 106:470–476. 20691303 10.1016/j.amjcard.2010.03.062

[53] Kao SC Pavlakis N Harvie R High blood neutrophil-to-lymphocyte ratio is an indicator of poor prognosis in malignant mesothelioma patients undergoing systemic therapy. *Clin Cancer Res* 2010; 16:5805–5813. 20956618 10.1158/1078-0432.CCR-10-2245

[54] Chua W Charles KA Baracos VE Neutrophil/lymphocyte ratio predicts chemotherapy outcomes in patients with advanced colorectal cancer. *Br J Cancer* 2011; 104:1288–1295. 21448173 10.1038/bjc.2011.100 PMC3078587

