# Supplementary material for: A Novel and Validated Inflammation-Based Score (IBS) Predicts Survival in Patients With Hepatocellular Carcinoma Following Curative Surgical Resection: A STROBE-Compliant Article
Source: Medicine (Baltimore). 2016 Feb 18;95(7):e2784. doi: 10.1097/MD.0000000000002784 (PMC4998627; doi:10.1097/MD.0000000000002784)

Figure S1. Receiving operating characteristic curves (ROC) of preoperative and postoperative NLR for predicting cancer related death. A cutoff value of 1.65 of preoperative and 3.65 of postoperative NLR was used to predict cancer related death (A) and recurrence (B) via ROC of patients with HCC after curative resection.


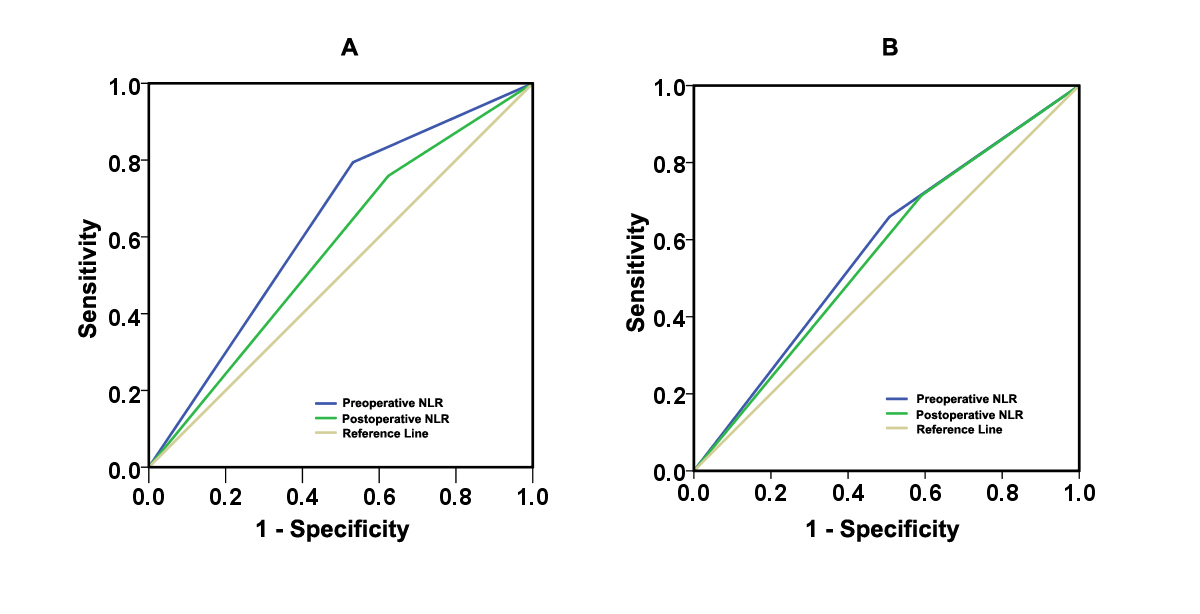


Figure S2. Kaplan - Meier survival curves for patients in validation cohort stratified by IBS. (A) overall survival; (B) recurrence free survival.


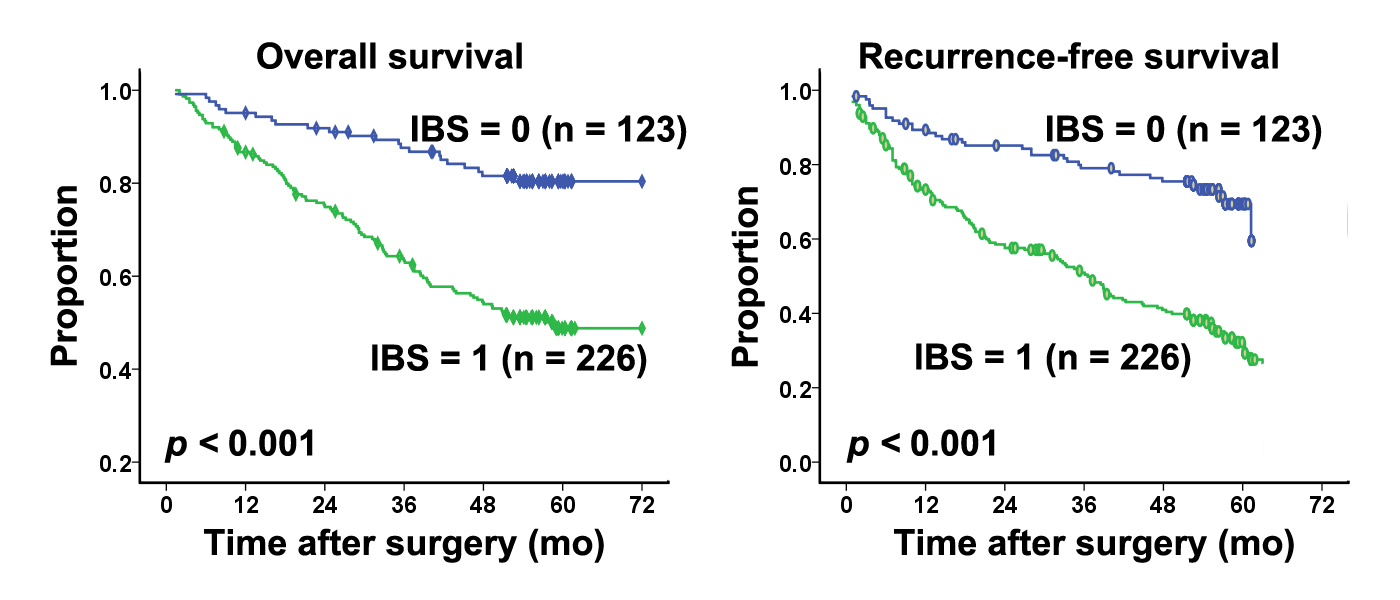

Supplement: Supplemental Digital Content [file medi-95-e2784-s001.doc]
